# HIV testing uptake, enablers, and barriers among African migrants in China: A nationwide cross-sectional study

**DOI:** 10.7189/jogh.12.11015

**Published:** 2022-12-17

**Authors:** Peizhen Zhao, Jiayu Wang, Brian J Hall, Kwame Sakyi, Mohamed Yunus Rafiq, Adams Bodomo, Cheng Wang

**Affiliations:** 1 STD Control Department, Dermatology Hospital, Southern Medical University, Guangzhou, China; 2 Southern Medical University Institute for Global Health, Guangzhou, China; 3 Department of Health Behavior, University of North Carolina at Chapel Hill, Chapel Hill, North Carolina, USA; 4 Center for Global Health Equity, New York University Shanghai, Shanghai, China; 5 Department of Public and Environmental Wellness, School of Health Sciences, Oakland University, Rochester, Michigan, USA; 6 Center for Learning and Childhood Development, Accra, Ghana; 7 Department of Anthropology, New York University Shanghai, Shanghai, China; 8 School of Liberal Arts, Xi'an University, Xi'an, China; 9 African Studies Department, University of Vienna, Vienna, Austria

## Abstract

**Background:**

African migrants in China face social, structural, and cultural barriers to human immunodeficiency virus (HIV) testing with scarce information on their HIV testing behaviours. This study estimated the prevalence of HIV testing and its social and behavioural correlates to understand how to better provide HIV testing services for African migrants living in China.

**Methods:**

We conducted a national cross-sectional survey among adult African migrants who lived in China for more than one month between January 19 to February 7, 2021. The survey was disseminated online through six African community organizations and via participant referrals. We collected data on HIV testing behaviours and history of HIV testing, social, and cultural factors and applied univariate and multivariable logistic regression to identify testing correlates.

**Results:**

Among a total of 1305 participants, 72.9% (n = 951/1305) tested for HIV during their stay in China and yielded a self-reported HIV prevalence of 0.4% (n = 4/951). The most common reason for HIV testing was to comply with Chinese residence policy requirements (88.5%, n = 842/951); for not testing was “no need to be tested” (79.4%, n = 281/354). We found most African migrants have experienced low acculturation stress (54.5%, n = 750/1305), low social discrimination (65.6%, n = 856/1305), have a moderate stigma towards HIV (54.3%, n = 709/1305), and low community engagement around sexual health and HIV topics. In multivariable analysis, African migrants who were students (adjusted odds ratio (aOR) = 3.36, 95% CI = 2.40-4.71), living in student dormitories (aOR = 3.86, 95% CI = 1.51-9.84), received health services in China in past year (aOR = 1.67, 95% CI = 1.25-2.23), had lifetime sexually transmitted infections (STI) testing (aOR = 1.95, 95% CI = 1.23-3.10), had HIV testing before coming to China (aOR = 13.56, 95% CI = 9.36-19.65), and those engaged in community discussions of HIV and sexual health (aOR = 2.77, 95% CI = 1.31-5.83) were more likely to test for HIV in China.

**Conclusions:**

Despite 73% of African migrants having tested for HIV in China, there are unmet needs and barriers identified in our study, such as language barriers. Access to HIV knowledge and testing services were the most important enablers for testing, including studentship, past STI/HIV testing, and community discussion on sexual health. Culturally appropriate and community-based outreach programs to provide information on HIV and testing venues for African migrants might be helpful to promote testing uptake.

Africans are among the fastest-growing migrant populations worldwide, transitioning from historically high-income destinations (HICs) to low- and middle-income countries (LMICs) [1,2]. 31% (12 millions) of African migrants moved to LMICs in 2019 compared with 17% in 2010, with the Asian continent quickly becoming the second largest recipient of African migrants after Europe [1,3]. Increasing numbers of Africans are migrating and residing in China due to increased trade and economic relations between the regions [4]. International evidence shows that migrants were at higher risk for human immunodeficiency virus (HIV) infections and faced additional structural and social challenges to access local health services [3,5-10]. However, there is scarce data on African migrants’ HIV testing behaviours and sexual health needs in China or in other LMICs to inform public health authorities on how best to support this population.

Currently, there are an estimated 200 000 to 450 000 Africans living in China including both students and workers [11-14]. Most African migrants self-identify as “traders/businessmen” or “students” and reside in China on short-term and long-term stays [13]. African businessmen usually travel frequently between their home country and destination cities in China. In 2016, around 62 000 African students were studying in China, with an annual increase of 24% [12]. African students are the fastest growing group among foreign students [12]. Interpersonal and cultural challenges, varying expectations of medical care, logistical problems at hospitals, and language issues were expressed as barriers to African migrants’ health care-seeking processes in China [15]. Since 2013, Chinese policy had required all foreigners who apply for visas longer than one year to submit HIV testing records in order to obtain the visa, regardless of the test results; though there are no mandates at the national level to get tested for HIV once foreigners enter China [16,17].

To date, there is little data on African migrants’ HIV testing behaviour, needs, and barriers in China. Given increasing globalization, more understanding of HIV testing and service utilization among African migrants is necessary and beneficial to both the local and global context. European studies found African migrants were at higher risk for HIV infection with lower rates of HIV testing compared with local residents, calling for tailored interventions to support this population [18-20]. A Belgian study found a high HIV prevalence of 4.2%-5.9% in sub-Saharan African migrants [19], yet due to cultural, financial, and structural barriers, they often presented to hospitals later when infected with HIV [3-9]. Delays in diagnosis and treatment can result in higher morbidity and mortality and increased HIV transmission risks in the community [21-23]. European studies found knowledge of HIV and high-risk behaviours, access to primary care, risky sexual behaviours, and non-stigma toward HIV was associated with increased HIV testing [18,20,24].

Evidence from HICs suggests that HIV stigma reduction, risk awareness education, strengthened linkage to care, and structural inclusion in health care would promote HIV testing in African migrants [5]. However, HIV testing behaviours and challenges can be inherently different in resource-limited settings than in HICs. Databased interventions addressing the unique needs of African migrants in China and other LMICs are urgently needed to fill the gap in the literature. This study aims to examine HIV testing experiences and its determinants among African migrants in China.

## METHODS

### Study design and participants

We conducted a national online cross-sectional survey between January 19 and February 7, 2021, using WenJuanXing online software (Changsha Haoxing Information Technology, China), a widely used online survey software in China. Individuals were deemed eligible if they self-identified as an African or of African parentage, aged more than 18 years old, cumulatively lived in China for more than one month, and were able to provide informed consent. All eligible participants received 5 US$ after completing the survey.

### Sample size

The primary outcome of this study was the HIV testing rate in China. A Chinese previous study reported a testing rate of 47.8% for HIV among African migrants [25]. We applied two-sided confidence intervals (CI) for one proportion method to estimate a sample size of 1096 for this study to produce a two-sided 95% CI and a width of 0.060.

### Data collection

The survey questionnaire was created in English in consultation with community-based organization stakeholders, policymakers, and experts on international migrants and HIV/sexually transmitted infections (STI) prevention. The questionnaires were piloted among 20 African migrants to ensure they were clear and comprehensible for the intended participants. The pilot data were excluded from the final data.

We used a peer-driven method for data collection. We first identified six active African community organization leaders (communities from Zimbabwe, Nigeria, Zambia, Tanzania, and Ghana) in China to facilitate initial study recruitment, where they mobilized peer networks to disseminate online recruitment to potential participants through WeChat (a widely-used Chinese messaging app). Through the link in WeChat, study participants access the questionnaire hosted on Wenjuanxing software. We did not collect any identifiable data and all responses were kept confidential. Second, participants were encouraged to recruit other eligible individuals to our questionnaire and informed that they would receive 2 US$ for each effective referral.

Participation in this survey was completely voluntary. Participants were informed it would take 20 minutes to complete the survey and that all responses would be kept confidential. Study individuals clicked “agree to participate” and signed the electronic informed consent if they were willing to participate in this study. To minimize repetitive recruitment, we only allowed the survey link to be accessed once by one IP address, phone, or WeChat account.

### Outcomes and exploratory variables

The primary outcome of interest is HIV testing or not while in China. Other HIV testing information collected included: the place of HIV counselling/ testing in China (public hospital/STI specialist hospital, private hospital, blood centre); latest HIV testing results (positive, negative, never got results); reasons for having HIV test (Chinese residence policy requirements, want to know my HIV status, enlightenment of publicity and education); and reason for not having HIV test (no need to be tested, language communication barriers, I don't know where to test).

Social demographic information collected included: gender (male, female), age (years), marital status (never married, ever married /engaged/ widowed/ divorced), highest educational attainment (high school or below, some college, bachelor or above), annual income (USD), religion, reasons for migration, cumulative stay in China, living arrangement in China and health insurance. Sexual behaviours information collected included: types of sexual partners, commercial sexual activities, and consistent condom use in sexual activities. Consistent condom use was defined as always using condoms when engaged in sexual activities.

Acculturative stress was measured by nine items, with each item coded 0 for no and 1 for yes, adapted from the National Latino and Asian American Study (Table S1 in the **Online Supplementary Document**) [26]. One example item is “Do you feel guilty for leaving family or friends in your country of origin”. The total scores ranged from 0 to 9 with 3 categories: low (0-3), moderate (4-6), and high (7-9). Higher score indicates greater self-reported acculturative stress. The Cronbach’s α of the acculturative stress scale in this study was 0.546.

Discrimination was measured by ten items, with each item scored 0-5 on a Likert-type scale (never, less than once a year, few times a year, few times a month, at least once a week, and almost every day), adapted from the Detroit Area Study (Table S2 in the **Online Supplementary Document**) [27]. One example is “Have you ever been treated with less courtesy while in China”. The total scores ranged from 0 to 50 with 3 categories: low (0-20), moderate (21-40), and high (41-50). A higher score indicates greater self-reported daily discrimination. The Cronbach’s α of the discrimination scale in this study was 0.930.

Anticipated HIV stigma was measured by seven items, with each item scored 0-3 on a Likert-type scale (strongly disagree, disagree, agree and strongly agree), developed by Golub and Gamarel among men who have sex with men in New York (Table S3 in the **Online Supplementary Document**) [28]. One example item is “If I had HIV, I'd worry about people discriminating against me”. The total scores ranged from 0 to 21 with 3 categories: low (0-8), moderate (9-16), and high (17-21). A higher score indicates a greater self-reported anticipated HIV stigma. The Cronbach’s α of the anticipated HIV stigma scale in this study was 0.879.

Community engagement on sexual and HIV topics was measured by six items that elicited participants’ awareness and advocacy involvement of HIV and sexual health, and each item was coded 0 for No and 1 for Yes. (Table S4 in the **Online Supplementary Document**) [29]. One example item is “Have you ever participated in online forums or discussions on social media (i.e., WhatsApp, WeChat, Weibo, Twitter, or other online communities) about sexual health, condom use, or HIV/STI testing or related services?” The total scores ranged from 0 to 6 with 3 categories: low (0-2), moderate (3-4), and high (5-6). A higher score indicated greater self-reported community engagement. The Cronbach’s α of community engagement scale in this study was 0.690.

### Statistical analysis

From the cross-sectional data, we described the socio-demographics, sexual behaviours, HIV testing, acculturative stress, discrimination, HIV stigma, and community engagement of African migrants in China. Categorical parameters were presented as the number (percentage) of participants. All the continuous data have been tested for normality using Kolmogorov-Smirnov method. We found that all the continuous data were normally distributed. Continuous data were expressed as mean ± standard deviation. The χ^2^ test was used to compare categorical variables. The difference in acculturative stress, discrimination, anticipated HIV stigma, and community engagement between the HIV testing subgroup and non-HIV testing subgroup in China was assessed with *t* test.

Univariable and multivariable logistic regression was conducted to explore socio-demographic, sexual behavioural factors, acculturative stress, discrimination, anticipated HIV stigma, and community engagement association with HIV testing. In the multivariable model, we adjusted for gender, age, legal marital status, highest educational attainment, annual income, and religion as these were considered non-changeable demographic features. In subgroup analysis comparing mandatory HIV testers and voluntary HIV testers, the same methods were used to compare socio-demographics and identify correlates for mandatory HIV testing. All analyses were conducted using SAS (V9.4, SAS Institute Inc., Cary, NC). All the results are deemed to be statistically significant when *P* ≤ 0.05.

## RESULTS

**Figure 1:**
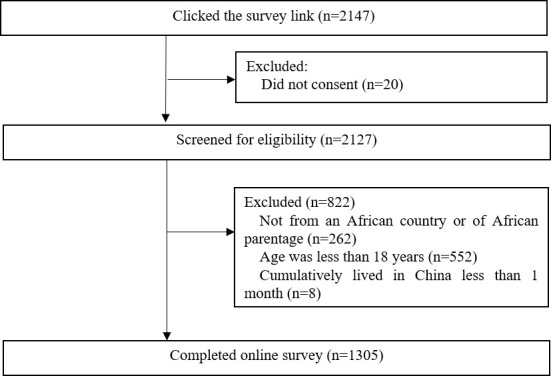
Study participants recruitment flowchart.

The survey platform was accessed by 2147 individuals, where twenty individuals did not provide informed consent and 822 did not meet the eligibility criteria (262 were not from an African country or of African parentage, 552 were younger than 18 years old and 8 did not cumulatively live in China for one month or longer) (**Figure 1**). A total of 1305 individuals were enrolled and completed the online survey.

These individuals lived in 98 cities in 26 provinces and regions of China and were originally from 51 countries in Africa, including Zimbabwe (33%), Nigeria (11%), Zambia (10%), Tanzania (9%), Ghana (7%), etc. (Table S5, Table S6 and Figure S1 in the **Online Supplementary Document**). 951 (72.9%) individuals had tested for HIV during their stay in China, with 4 individuals (0.4%, n = 4/951) reporting a positive result in their latest HIV testing.

### Social demographics and sexual behaviours

The majority of participants were male (64.6%, n = 843/1305), between 18 and 25 years old (73.8%, n = 963/1305), never married (93.1%, n = 1215/1305), finished high school or higher (77.3%, n = 1009/1305), were Christian (80.2%, n = 1047/1305), had health insurance in China (87.5%, n = 1142/1305), and had cumulatively stayed in China over one year (93.3%, n = 1217/1305). Over half of the study participants migrated to China for educational activities (59.2%, n = 773/1305). About half of the participants had an annual income of less than US$ 2000(45.5%, n = 594/1305) (**Table 1**).

**Table 1:** Social demographic and sexual behavioural characteristics among African migrants in China, 2021 (n = 1305)

Variables	n (%)	HIV testing in China		χ^2^	*P*
		**Ever test**	**Never test**		
**Total**	1305	951 (72.9)	354 (27.1)		
**Gender**				10.03	0.002
Male	843 (64.6)	590 (62.0)	253 (71.5)		
Female	462 (35.4)	361 (38.0)	101 (28.5)		
**Age (years)**				4.21	0.122
18-25	963 (73.8)	716 (75.3)	247 (69.8)		
26-35	322 (24.7)	222 (23.3)	100 (28.2)		
≥36	20 (1.5)	13 (1.4)	7 (2.0)		
**Legal marital status**				0.01	0.919
Never married	1215 (93.1)	885 (93.1)	330 (93.2)		
Ever married/engaged/widowed/divorced	90 (6.9)	66 (6.9)	24 (6.8)		
**Highest educational attainment**				9.21	0.010
High school or below	296 (22.7)	235 (24.7)	61 (17.2)		
Some college	392 (30.0)	285 (30.0)	107 (30.2)		
Bachelor’s or higher	617 (47.3)	431 (45.3)	186 (52.6)		
**Annual income (US$)**					
<2000	594 (45.5)	456 (48.0)	138 (39.0)	20.46	<0.001
2000-5000	338 (25.9)	253 (26.6)	85 (24.0)		
5000-10000	123 (9.4)	87 (9.1)	36 (10.2)		
>10000	250 (19.2)	155 (16.3)	95 (26.8)		
**Religion**				67.74	<0.001
Christianity	1047 (80.2)	812 (85.4)	235 (66.4)		
Muslim	176 (13.5)	85 (8.9)	91 (25.7)		
Other	11 (0.8)	8 (0.8)	3 (0.9)		
None	71 (5.4)	46 (4.8)	25 (7.1)		
**Reasons for migration**				72.71	<0.001
Business	411 (31.5)	241 (25.3)	170 (48.0)		
Study	773 (59.2)	629 (66.1)	144 (40.7)		
Employment	88 (6.8)	58 (6.1)	30 (8.5)		
Tourism/visiting relatives	33 (2.5)	23 (2.4)	10 (2.8)		
**Cumulative stay in China**				2.40	0.301
1-6 mo	37 (2.8)	25 (2.6)	12 (3.4)		
7-12 mo	51 (3.9)	33 (3.5)	18 (5.1)		
One year and above	1217 (93.3)	893 (93.9)	324 (91.5)		
**Living arrangements in China**				43.70	<0.001
Hotel	44 (3.4)	21 (2.2)	23 (6.5)		
Guest apartment	46 (3.5)	32 (3.4)	14 (4.0)		
Purchased apartment	20 (1.5)	10 (1.0)	10 (2.8)		
Rental apartment	514 (39.4)	346 (36.4)	168 (47.5)		
Staff/student dormitory	663 (50.8)	529 (55.6)	134 (37.8)		
No regular residence	18 (1.4)	13 (1.4)	5 (1.4)		
**Health insurance in China**				0.51	0.476
Yes	1142 (87.5)	836 (87.9)	306 (86.4)		
No	163 (12.5)	115 (12.1)	48 (13.6)		
**Have you had sex during your stay in China**				2.14	0.143
Yes	488 (37.4)	367 (38.6)	121 (34.2)		
No	817 (62.6)	584 (61.4)	233 (65.8)		
Regular partner*				6.73	0.010
Yes	444 (91.0)	341 (92.9)	103 (85.1)		
No	44 (9.0)	26 (7.1)	18 (14.9)		
Casual partner*				1.37	0.241
Yes	58 (11.9)	40 (10.9)	18 (14.9)		
No	430 (88.1)	327 (89.1)	103 (85.1)		
**Number of sexual partners**					
	2.1 ± 3.0	1.9 ± 2.6	2.3 ± 3.9	1.22	0.224
**Ever had commercial sexual activities**					
Yes	17 (1.3)	11 (1.2)	6 (1.7)	0.58	0.446
No	1288 (98.7)	940 (98.8)	348 (98.3)		
**Consistently used condoms in sexual activities**				3.02	0.082
Yes	257 (52.7)	185 (50.4)	72 (59.5)		
No	231 (47.3)	182 (49.6)	49 (40.5)		
**Injected drugs in the past year**				2.67	0.102
Yes	10 (0.8)	5 (0.5)	5 (1.4)		
No	1295 (99.2)	946 (99.5)	349 (98.6)		
**Have you received health services in China in the past year†**				8.90	0.003
Yes	445 (34.1)	347 (36.5)	98 (27.7)		
No	860 (65.9)	604 (63.5)	256 (72.3)		
**Have you ever had any STD testing other than HIV testing‡**				10.76	0.001
Yes	155 (11.9)	130 (13.7)	25 (7.1)		
No	1150 (88.1)	821 (86.3)	329 (92.9)		
**Had HIV testing before coming to China**				281.14	<0.001
Yes	1097 (84.1)	898 (94.4)	199 (56.2)		
No	208 (15.9)	53 (5.6)	155 (43.7)		

HIV – human immunodeficiency virus, STD – sexually transmitted diseases

*Participants had sex during the stay in China.

†Health services other than HIV testing.

‡STD testing includes testing for syphilis, gonorrhoea, chlamydia, human papilloma virus, and herpes simplex virus.

About one-third (37.4%, n = 488/1305) of the participants had sex during their stay in China, of whom 91.0% (n = 444/488) had sex with regular partners and 11.9% (n = 58/488) reported having sex with casual partners. 52.7% (n = 257/488) of the participants used condoms consistently in sexual activities and 1.3% (n = 17/1305) had commercial sex. For health service utilization, 34.1% (n = 445/1305) received health services in China in the past year, and 11.9% (n = 155/1305) had lifetime STD testing before. Additionally, a majority of the participants (84.1%, n = 1097/1305) had HIV testing before coming to China (**Table 1**).

### HIV testing utilization

Among the 951 individuals who had tested for HIV in China, the most common place for HIV testing was in public hospital/STI specialist hospital (61.5%, n = 585/951), followed by private hospitals (23.7%, n = 225/951) and blood centre (10.6%, n = 101/951). The main reason for HIV testing in China was to comply with Chinese residence policy requirements (88.5%, n = 842/951) and wanting to know their HIV status (20.2%, n = 192/951) **(****Table 2****)**. Disaggregated by sexual activity status, 75.2% of those who had sex in China tested for HIV, while 71.5% of those who never had sex in China tested for HIV.

**Table 2:** Point of service utilization and reasons for HIV testing

Variables	n	%
**Place of HIV testing in China (n = 951)**		
Public hospital/STD specialist hospital	585	61.5
Private hospital	225	23.7
Blood centre	101	10.6
Community health centre	86	9.0
Used a self-test kit	47	4.9
CDC (voluntary counselling HIV test site)	17	1.8
Community-based organization	16	1.7
Other (immigration hospital, school hospital)	50	5.3
**Latest HIV testing results (n = 951)**		
Positive	4	0.4
Negative	935	98.3
Never got results	12	1.3
**Reasons for having HIV tests (n = 951)**		
Chinese residence policy requirements	842	88.5
Want to know my HIV status	192	20.2
Enlightenment of publicity and education	36	3.8
Doctor's request/recommendation	28	2.9
Partners recommendation	27	2.8
High-risk sexual contact	4	0.4
I had symptoms I was worried it was due to HIV	2	0.2
Drug use	1	0.1
Other (school requirements, health checks)	24	2.5
**Reasons for not having HIV tests (n = 354)**		
No need to be tested	281	79.4
Language communication barriers	51	14.4
I don't know where to test	35	9.9
I am worried that the cost is too high	28	7.9
I am too busy to spare time to test	16	4.5
Fear of exposing my privacy causes discrimination	8	2.3
I am afraid to go to the health facility for testing	5	1.4
I am afraid of being deported from the country with HIV positive	4	1.1
I have no medical insurance	3	0.9
Other	3	0.9

HIV – human immunodeficiency virus, STD – sexually transmitted diseases, CDC – centres for disease control and prevention

Among those not tested (n = 354), the most common reason was “no need to be tested” (79.4%, n = 281/354), followed by language communication barriers (14.4%, n = 51/354) and “don't know where to test” (9.9%, n = 35/354) (**Table 2**).

### Acculturation, stigma, and community engagement

#### Acculturative stress

A small percentage of participants experienced high acculturative stress (4.3%, n = 56/1305), over a third experienced moderate acculturative stress (38.2%, n = 499/1305), and over half had low acculturative stress (54.5%, n = 750/1305). There was no significant association between acculturative stress and having an HIV test in China (*P* = 0.051).

#### Feeling discriminated against as a foreigner in China

3.8% (n = 49/1305) of participants experienced a high level of discrimination as a foreigner in China, a third reported moderate discrimination (30.6%, n = 400/1305), and the majority experienced no or low level of discrimination (65.6%, n = 856/1305). Ever had HIV testing in China was found to be associated with feeling discriminated against as a foreigner in China (*P* = 0.029).

#### Anticipated HIV stigma

A minority of participants displayed a high level of stigma towards HIV (13.6%, n = 178/1305), most participants expressed a moderate level of stigma towards HIV (54.3%, n = 709/1305), and about one-third of participants showed a low level of HIV stigma (32.0%, n = 418/1305). There was no statistically significant association between the anticipated HIV stigma of the participants and having an HIV test in China (*P* = 0.385).

#### Community engagement on HIV and sexual health

Few (4.8%, n = 62/1305) participants actively engaged in community discussion around HIV and sexual health, 12.8% (n = 167/1305) were moderately involved with these discussions, and the majority of participants had low or no involvement in a community discussion on HIV and sexual health (82.4%, n = 1076/1305). HIV testing in China was found to be associated with a higher score of community engagement on this topic (*P* < 0.001) (**Table 3**).

**Table 3:** Acculturation, discrimination, HIV stigma and community engagement among African migrants in China, 2021 (n = 1305)

Variables	HIV testing in China				
	**Total**	**Ever test**	**Never test**	***t*/χ^2^**	***P***
**Acculturative stress**				0.68	0.713
High	56 (4.3)	40 (4.2)	16 (4.5)		
Moderate	499 (38.2)	370 (40.0)	129 (37.5)		
Low	750 (54.5)	541 (58.4)	209 (60.8)		
Mean ± SD	3.2 ± 1.8	3.3 ± 1.8	3.1 ± 2.0	1.95	0.051
**Feeling discriminated against as a foreigner in China**				0.59	0.742
High	49 (3.8)	36 (3.8)	13 (3.7)		
Moderate	400 (30.6)	297 (31.2)	103 (29.1)		
Low	856 (65.6)	618 (65.0)	238 (67.2)		
Mean ± SD	16.0 ± 12.6	16.5 ± 12.5	14.8 ± 12.7	2.18	0.029
**Anticipated HIV stigma**				0.85	0.654
High	178 (13.6)	127 (13.4)	51 (14.4)		
Moderate	709 (54.3)	524 (55.1)	185 (52.3)		
Low	418 (32.0)	300 (31.5)	118 (33.3)		
Mean ± SD	10.8 ± 5.1	10.8 ± 4.9	10.6 ± 5.6	0.87	0.385
**Community engagement on HIV and sexual health**				18.34	<0.001
High	62 (4.8)	53 (5.6)	9 (2.5)		
Moderate	167 (12.8)	140 (14.7)	27 (16.2)		
Low	1076 (82.4)	758 (79.7)	318 (89.8)		
Mean ± SD	1.3 ± 1.5	1.4 ± 1.5	0.9 ± 1.3	6.09	<0.001

HIV – human immunodeficiency virus, SD – standard deviation

#### Regression analysis: Correlates of HIV testing

After adjusting for gender, age, marital status, educational attainment, annual income, and religion, multivariable logistic regression analysis indicated that African migrants who migrated to China for study (adjusted odds ratio (aOR) = 3.36, 95% CI = 2.40-4.71), lived in staff/student dormitories compared with those who purchased an apartment (aOR = 3.86, 95% CI = 1.51-9.84), received health services in China in past year (aOR = 1.67, 95% CI = 1.25-2.23), had lifetime STI testing (aOR = 1.95, 95% CI = 1.23-3.10), had HIV testing before coming to China (aOR = 13.56, 95% CI = 9.36-19.65), and those had a higher level of engagement in the community on HIV and sexual health topics (aOR = 2.77, 95% CI = 1.31-5.83) were more likely to have an HIV test in China (**Table 4**).

**Table 4:** Factors correlated with HIV testing among African migrants in China, 2021 (n = 1305)

Variables	HIV test% (n)	cOR (95%CI)	*P*	aOR (95%CI)§	*P*
**Gender**					
Male	70.0 (590/843)	Ref		-	-
Female	78.1 (361/462)	1.53 (1.18-2.00)	0.002	-	-
**Age (years)**					
16-25	74.4 (716/963)	Ref		-	-
26-35	68.9 (222/322)	0.77 (0.58-1.01)	0.059	-	-
≥36	65.0 (13/20)	0.64 (0.25-1.62)	0.348	-	-
**Legal marital status**					
Never married	72.8 (885/1215)	Ref		-	-
Ever married/engaged	73.3 (66/90)	1.03 (0.63-1.66)	0.919	-	-
**Highest educational attainment**					
High school or below	79.4 (235/296)	Ref		-	-
Some college	72.7 (285/392)	0.69 (0.48-0.99)	0.044	-	-
Bachelor’s or higher	69.9 (431/617)	0.60 (0.43-0.84)	0.003	-	-
**Annual income (US$)**					
<2000	76.8 (456/594)	Ref		-	-
2000-5000	74.9 (253/338)	0.90 (0.66-1.23)	0.51	-	-
5000-10000	70.7 (87/123)	0.73 (0.48-1.13)	0.156	-	-
>10000	62.0 (155/250)	0.49 (0.36-0.68)	<0.001	-	-
**Religion**					
Christianity	77.6 (812/1047)	1.88 (1.13-3.12)	0.015	-	-
Islam	48.3 (85/176)	0.51 (0.29-0.90)	0.020	-	-
Other	72.7 (87/11)	1.45 (0.45-5.96)	0.607	-	-
None	64.8 (46/71)	Ref		-	-
**Reasons for migration**					
Business	58.6 (241/411)	Ref		Ref	
Study	81.4 (629/773)	3.08 (2.36-4.02)	<0.001	3.36 (2.40-4.71)	<0.001
Employment	65.9 (58/88)	1.36 (0.84-2.21)	0.208	1.22 (0.74-2.03)	0.434
Tourism/visiting relatives	63.7 (23/33)	1.62 (0.75-3.50)	0.217	1.75 (0.77-3.95)	0.181
**Cumulative stay in China**					
1-6 mo	67.6 (25/37)	Ref		Ref	
7-12 mo	64.7 (33/51)	0.88(0.36-2.16)	0.78	0.88(0.34-2.23)	0.779
One year and above	73.4 (893/1217)	1.32(0.66-2.66)	0.433	1.27(0.61-2.62)	0.524
**Living arrangements in China**					
Purchased apartment	50.0 (10/20)	Ref		Ref	
Hotel	47.7 (21/44)	0.91 (0.32-2.63)	0.866	0.96 (0.32-2.88)	0.945
Guest apartment	69.6 (32/46)	2.29 (0.78-6.72)	0.133	2.29 (0.75-7.05)	0.148
Rental apartment	67.3 (346/514)	2.06 (0.84-5.04)	0.114	2.25 (0.90-5.67)	0.084
Staff/student dormitory	79.8 (529/663)	3.95 (1.61-9.68)	0.003	3.86 (1.51-9.84)	0.005
No fixed residence	72.2 (13/18)	2.60 (0.67-10.07)	0.167	2.52 (0.61-10.45)	0.203
**Health insurance in China**					
Yes	73.2 (836/114)	1.14 (0.79-1.64)	0.476	1.15 (0.79-1.69)	0.465
No	70.6 (115/163)	Ref		Ref	
**Have you had sex during your stay in China**					
Yes	75.2 (367/488)	Ref		Ref	
No	71.5 (584/817)	0.83 (0.64-1.07)	0.144	0.88 (0.67-1.15)	0.333
Regular partner*					
Yes	76.8 (341/444)	2.29 (1.21-4.35)	0.011	2.01 (0.97-4.17)	0.061
No	59.1 (26/44)	Ref		Ref	
Casual partner*					
Yes	69.0 (40/58)	0.70 (0.39-1.27)	0.243	0.91 (0.46-1.80)	0.788
No	76.1 (327/430)	Ref		Ref	
**Number of sexual partners**					
	1.9 ± 2.6	0.96 (0.90-1.03)	0.245	1.01 (0.94-1.10)	0.757
**Consistently used condoms in sexual activities**					
Yes	72.0 (185/257)	Ref		Ref	
No	78.8 (182/231)	1.45 (0.95-2.19)	0.083	1.46 (0.93-2.28)	0.102
**Ever had commercial sexual activities**					
Yes	64.7 (11/17)	0.68 (0.25-1.85)	0.448	0.90 (0.31-2.6)	0.839
No	73.0 (940/1288)	Ref		Ref	
**Injected drugs in the past year**					
Yes	50.0 (55/10)	0.34 (0.10-1.20)	0.094	1.01 (1.00-1.02)	0.137
No	73.1 (946/129)	Ref		Ref	
**Have you received health services in China in the past year†**					
Yes	78.0 (347/445)	1.50 (1.15-1.96)	0.003	1.67 (1.25-2.23)	0.005
No	70.2 (604/860)	Ref		Ref	
**Have you ever had any STD testing other than HIV testing‡**					
Yes	83.9 (130/155)	2.08 (1.33-3.26)	0.001	1.95(1.23-3.10)	0.005
No	62.9 (821/1150)	Ref		Ref	
**Had HIV testing before coming to China**					
Yes	81.9 (898/1097)	13.19 (9.32-18.78)	<0.001	13.56 (9.36-19.65)	<0.001
No	25.5 (53/208)	Ref		Ref	
**Acculturative stress**					
High	71.4 (40/56)	0.97 (0.53-1.76)	0.910	1.05 (0.56-1.98)	0.879
Moderate	74.2 (370/499)	1.11 (0.86-1.43)	0.433	1.06 (0.82-1.39)	0.648
Low	72.1 (541/750)	Ref		Ref	
**Feel discriminated as a foreigner in China**					
High	73.5 (36/49)	1.07 (0.56-2.05)	0.847	0.90 (0.46-1.76)	0.758
Moderate	74.2 (297/400)	1.11 (0.85-1.45)	0.446	1.07 (0.81-1.42)	0.649
Low	72.2 (618/856)	Ref		Ref	
**Anticipated HIV stigma**					
High	71.4 (127/178)	0.98 (0.66-1.44)	0.917	1.03 (0.69-1.56)	0.875
Moderate	73.9 (524/709)	1.11 (0.85-1.46)	0.435	1.14 (0.85-1.51)	0.381
Low	71.8 (300/418)	Ref		Ref	
**Community engagement**					
High	85.5 (53/62)	2.47 (1.21-5.07)	0.014	2.77 (1.31-5.83)	0.008
Moderate	83.8 (140/167)	2.18 (1.41-3.35)	<0.001	2.14 (1.37-3.36)	<0.001
Low	70.5 (758/1075)	Ref		Ref	

HIV – human immunodeficiency virus, aOR – adjusted odds ratio, cOR – crude odds ratio, CI – confidence interval

*Participants had sex during the stay in China.

†Health services other than HIV testing.

‡STD testing includes testing for syphilis, gonorrhoea, chlamydia, human papilloma virus, and herpes simplex virus.

§Adjusted for gender, age, legal marital status, highest educational attainment, annual income, and religion.

Subgroup analysis between mandatory testers (n = 842) and voluntary testers (n = 109) found that those who tested for HIV mandatorily were younger, living in staff/student dormitories, and have health insurance in China; those who tested for HIV voluntarily were more likely to have had a sexual contact in China. The two groups also differed in annual income levels (Table S7 in the **Online Supplementary Document**). In multivariate analysis, living in staff/student dormitories (aOR = 4.23, 95% CI = 1.46-12.27) and did not have sexual contact in China (aOR = 1.55, 95% CI = 1.03-2.34), were associated with testing for HIV for mandatory reasons. Individuals who experienced a moderate level of acculturation stress (aOR = 0.37, 95% CI = 0.16-0.84), and had a moderate level of engagement in sexual health and HIV topics in the community (aOR = 0.40, 95% CI = 0.19-0.85), compared to those that had a low level of acculturation stress were associated with a lesser likelihood of testing for HIV for mandatory reasons, in other words, testing more likely due to voluntary reasons (Table S8 in the **Online Supplementary Document****)**.

## DISCUSSION

Our study provides the first national cross-sectional estimates of HIV testing uptake and sexual behaviours of African migrants in China. We found that 72.9% African migrants tested for HIV in China, comparable to 58.3%-89.6% African migrants tested in European countries [18-20,24]. 75.2% of sexually active migrants tested for HIV, slightly higher than those not having sex in China (71.5%). Self-reported HIV prevalence is 0.4%, lower than the 4.2%-5.9% found among African migrants in Europe and the prevalence in their home countries ranging from 1.4% to 12.9%, while 10 times the estimated prevalence of 0.037% in general Chinese population [19,30,31]. Our finding stresses the relevance to continually promote HIV testing uptake for African migrants to diagnose those infected with HIV and link them to care in a timely manner.

Since 2013, Chinese policy removed negative HIV testing result as a visa requirement for foreigners entering the border but have kept a proof of HIV test as a required document in the visa application materials. Once foreigners entered China, there are no mandates at the national level to get tested for HIV during their stay [16,17]. Among those never tested in China, the most common reason reported was “no need to be tested” (79.4%), despite the fact that only 53% of participants who have had sex reported consistent use of condoms, and 12% had sex with casual partners. Our findings of low risk perception as a dominant reason for not testing echoes with findings from Zimbabwe and Europe, where 33%-48% African migrants who had sex with non-steady partners consistently used condom and “not necessary to get tested” continuedly being expressed as a top reason for not testing [18,20,32]. Additionally, language barrier (14.4%), and lack of information on testing venues (9.9%) were also identified as barriers for testing, suggesting that support for more accessible and visible routes for African migrants to test for HIV is needed, for example, providing multiple language services or information brochures at public and community health facilities. Offering HIV testing to African migrants in general medical practice was also found to be acceptable and feasible in European settings [33,34].

Successfully delivering HIV prevention efforts for African migrants will require expanding culturally and socially appropriate services. We observed a lower frequency of community engagement on sexual health and HIV than in a German study, where 57% of African migrants reported discussion of HIV within their community [16]. We did find that participating in community discussion around sexual health and HIV, moderately or frequently, was associated with doubled and tripled odds of testing for HIV, aligned with the German study which reported discussing HIV in the community increased HIV testing odds by 92% [20]. This might be because community discussion on such sexual health topics can disseminate accurate information and knowledge on HIV, which can improve testing uptake based on data from an African migrant community in Britain [35]. We also observed low-to-medium level of acculturation stress, social discrimination, and over half present moderate stigma towards HIV, consistent with findings from North America and UNAIDS [36,37]. Studies have shown that acculturation stress, social discrimination and HIV stigma were barriers for HIV testing [38-40], though they were not associated to HIV testing as hypothesized in our study.

African students are more likely to participate in HIV testing in China. This might be attributable to health requirement policies on campus and their better knowledge and education on HIV, which were found as promoting factors for HIV testing [35]. Compared with businessmen and workers, students may have better access to HIV-related information and testing, through school-based HIV advocacy and education events, self-testing kits on campus, and routine health examinations for students [41,42]. This alarms policy-makers of the current vacuum in HIV testing efforts to reach non-student migrants. For health service utilization, 87.5% of African migrants in our study had health insurance in China and 34.1% have received health care services in China in past year, which was associated with a 67% increase in likelihood of testing for HIV. Existing care utilization, including previous STI and HIV testing, may remove the initial logistical and psychological barriers for getting tested for HIV. The findings align with the experience of African migrants in 9 countries in Europe, where access to primary care being a strong enabler for HIV testing [18]. Considered as a whole, our findings highlight that access to accurate HIV knowledge and testing services can be important enablers to improve HIV testing uptake.

Our study has several limitations. The cross-sectional study design limits causal inference ability. Africans in China are a hard-to-reach population and there are no known registries to ensure a representative sample, the participants were recruited using non-probabilistic sampling method, which may result in sampling bias. However, we reached 26 out of 34 provinces in China and covered the most populous and economically developed regions to increase the representativeness of this sample. The questionnaire was in English language and all data collected in this study was self-reported by participants, which may be biased and not verified. We offered research subjects financial incentives for their participation in this study, which may lead to biased enrollment. Further studies to understand how enablers and barriers impact HIV testing uptake, and implementation science study to tailor efforts for migrant subgroups, such as students, businessmen, and seasonal migrants who face different social environment are in urgent need.

## CONCLUSIONS

Our study presents the first HIV testing prevalence among African migrants in China. Though 73% African migrants tested for HIV in China, unmet needs and barriers for testing were identified, such as language, lack of knowledge on testing venues, and potentially low risk perception. Access to accurate HIV knowledge and HIV testing services were the most important enablers for HIV testing uptake. Studentship, past STI/HIV testing, and community discussion on sexual health topics were promoting factors for HIV testing. Culturally appropriate and community-based outreach programs to facilitate knowledge sharing on HIV, sexual health, and testing access will be beneficial to promote testing and HIV prevention efforts for this population.

## Additional material


Online Supplementary Document
