# Supplementary material for: HIV testing uptake, enablers, and barriers among African migrants in China: A nationwide cross-sectional study
Source: J Glob Health. 2022 Dec 17;12:11015. doi: 10.7189/jogh.12.11015 (PMC9758700; doi:10.7189/jogh.12.11015)
Supplement: Online Supplementary Document [file jogh-12-11015-s001.pdf]

HIV testing uptake, enablers, and barriers among African migrants in China: A nationwide cross-sectional study

Peizhen Zhao, Jiayu Wang, Brian J. Hall, Kwame Sakyi, Mohamed Yunus Rafiq, Adams Bodomo, Cheng Wang

Table S1 Description on acculturative stress scale

Item	Yes *n* (%)	No *n* (%)
Do you feel guilty for leaving family or friends in your country of origin	214(16.4)	1091(83.6)
Do you feel that in China you have the respect you had in your country of origin	576(44.1)	729(55.9)
Do you feel that living out of your country of origin has limited your contact with family or friends	777(59.5)	528(40.5)
Do you find it hard interacting with others because of difficulties you have with the Chinese language	796(61.0)	509(39.0)
Do people treat you badly because they think you do not speak Chinese well or speak with an accent	319(24.4)	986(75.6)
Do you find it difficult to find the work you want because you are of Africa descent	780(59.8)	525(40.2)
Have you been questioned about your legal status	417(32.0)	888(68.1)
Do you think you will be deported if you go to a social or government agency	237(18.2)	1068(81.8)
Do you avoid seeking health services due to fear of immigration officials	118(9.0)	1187(91.0)

Table S2 Description on discrimination scale

Item	Almost everyday *n* (%)	At least once week *n* (%)	Few times month *n* (%)	Few times year *n* (%)	Less than once year *n* (%)	Never *n* (%)
Have you ever been treated with less courtesy while in China	118(9.0)	114(8.7)	272(20.8)	290(22.2)	132(10.1)	379(29.0)
Have you ever been treated with less respect than others while in China	115(8.8)	125(9.6)	239(18.3)	289(22.2)	172(13.2)	365(28.0)
Have you ever received poorer service than others at restaurants or stores while in China	76(5.8)	114(8.7)	190(14.6)	220(16.9)	174(13.3)	531(40.7)
Have you ever met people who act as if they think you are not smart while in China	124(9.5)	98(7.5)	206(15.8)	266(20.4)	155(11.9)	456(34.9)
Have you ever met people who act as if they are afraid of you while in China	253(19.4)	141(10.8)	239(18.3)	269(20.6)	127(9.7)	276(21.2)

Have you ever met people who act as if they think you are dishonest while in China	107(8.2)	97(7.4)	181(13.9)	211(16.2)	147(11.3)	562(43.1)
Have you ever met people who act as if you are not as good while in China	112(8.6)	115(8.8)	190(14.6)	238(18.2)	177(13.6)	473(36.3)
Have you ever been called names or insulted while in China	97(7.4)	86(6.6)	149(11.4)	195(14.9)	182(14.0)	596(45.7)
Have you ever been threatened or harassed while in China	52(4.0)	64(4.9)	103(7.9)	153(11.7)	138(10.6)	795(60.9)
Have you had problems seeing a doctor because of your skin color	43(3.3)	38(2.9)	60(4.6)	102(7.8)	81(6.2)	981(75.2)

Table S3 Description on anticipated HIV stigma scale

**Item**	**Strongly Disagree *n* (%)**	**Disagree *n* (%)**	**Agree *n* (%)**	**Strongly Agree *n* (%)**
If I had HIV, I'd worry about people discriminating against me.	155(11.9)	324(24.8)	546(41.8)	280(21.5)
If I got infected with HIV, no one would date or become involved with me.	175(13.4)	454(34.8)	427(32.7)	249(19.1)
If I got infected with HIV, no one would want to have sex with me.	150(11.5)	330(25.3)	447(34.3)	378(29.0)
If I got infected with HIV, I would work hard to keep my HIV status a secret.	268(20.5)	558(42.8)	312(23.9)	167(12.8)
If I got infected with HIV, I would feel set apart and isolated from the rest of the world.	230(17.6)	499(38.2)	379(29.0)	197(15.1)
If I got infected with HIV, I would feel I was not as good as a person as others.	288(22.1)	526(40.3)	316(24.2)	175(13.4)
I would feel ashamed of getting HIV.	184(14.1)	371(28.4)	470(36.0)	280(21.5)

Table S4 Description on community engagement scale

**Item**	**Yes *n* (%)**	**No *n* (%)**
Have you ever participated in online forums or discussions on social media (i.e., WhatsApp, WeChat, Weibo, Twitter, or other on-line communities) about sexual health, condom use, or HIV/STI testing or related services?	399(30.6)	906(69.4)
Are you aware of any community events promoting sexual health among African migrants in China?	232(17.8)	1073(82.2)
Have you ever helped organize a testing and/or awareness campaign (e.g. HIV, condom use, etc.) that promoted sexual health among African migrants during your stay in China?	127(9.7)	1178(90.3)
Have you ever volunteered at a health clinic or other location that provided sexual health services among African migrants during your stay in China?	109(8.4)	1196(91.7)
Have you ever encouraged any African migrants to get tested for HIV and/or another sexually transmitted infection during your stay in China?	561(43.0)	744(57.0)

Have you ever accompanied a friend or partner to a testing facility to get tested for HIV and/or another sexually transmitted infection during your stay in China?	262(20.1)	1043(79.9)

Table S5 Countries of origins of study participants (*N*=1305)

Country	Number	Percentage
Republic of Zimbabwe	434	33.3
Federal Republic of Nigeria	144	11.0
Republic of Zambia	129	9.9
United Republic of Tanzania	115	8.8
Republic of Ghana	89	6.8
Republic of Liberia	64	4.9
Federal Democratic Republic of Ethiopia	53	4.1
Republic of South Africa	32	2.5
Republic of Uganda	28	2.1
Republic of Rwanda	27	2.1
Republic of Cameroon	16	1.2
Arab Republic of Egypt	14	1.1
Republic of Botswana	13	1.0
Republic of the Congo	13	1.0
Republic of the Sudan	13	1.0
Republic of Burundi	11	0.8
Republic of Mali	11	0.8
Republic of Côte d'Ivoire	8	0.7
Republic of Malawi	8	0.6
Republic of Madagascar	7	0.5
Republic of Guinea	6	0.5
Kingdom of Lesotho	6	0.5
Gabonese Republic	5	0.4
Republic of Angola	4	0.3
Republic of Benin	4	0.3
Republic of Mozambique	4	0.3
People's Democratic Republic of Algeria	3	0.2
Democratic Republic of the Congo	3	0.2
Republic of Equatorial Guinea	3	0.2
State of Eritrea	3	0.2
Republic of the Gambia	3	0.2
Republic of Kenya	3	0.2
Kingdom of Morocco	3	0.2
Republic of Sierra Leone	3	0.2
Togolese Republic	3	0.2
Burkina Faso	2	0.2
Republic of Chad	2	0.2
Union of the Comoros	2	0.2
Republic of Namibia	2	0.2

Republic of Senegal	2	0.2
Republic of South Sudan	2	0.2
Republic of Seychelles	2	0.2
Central African Republic	1	0.1
Republic of Guinea-Bissau	1	0.1
Republic of the Niger	1	0.1
Tunisian Republic	1	0.1
Democratic Republic of Sao Tome and Principe	1	0.1
Federal Republic of Somalia	1	0.1

Table S6 Geographic distribution of study participants' residence in China

Province	Number	Percentage
Zhejiang	342	27.6
Hubei	129	10.4
Jiangsu	119	9.6
Liaoning	80	6.4
Jiangxi	64	5.2
Fujian	60	4.8
Guangdong	59	4.8
Shandong	46	3.7
Beijing	45	3.6
Sichuan	40	3.2
Jilin	29	2.3
Shanghai	27	2.2
Anhui	20	1.6
Hebei	20	1.6
Hunan	20	1.6
Guangxi	19	1.5
Hainan	18	1.5
Shaanxi	17	1.4
Tianjin	16	1.3
Henan	14	1.1
Hongkong	13	1.0
Chongqing	12	1.0
Shanxi	11	0.9
Yunnan	11	0.9
Gansu	5	0.4
Heilongjiang	5	0.4
Unknown	64	4.9
Total	1305	100

Table S7. Social demographic and sexual behavioral characteristics among African migrants who had HIV testing in China by mandatory and voluntary testing, 2021 (*N*=951)

Variables	*N* (%)	HIV Testing in China		χ^2	*P*
		Voluntary testing	Mandatory testing		
**Total**	951	109(11.5)	842(88.5)		
**Gender**				0.12	0.733
Male	590(62.0)	66(60.6)	524(62.2)		
Female	361(38.0)	43(39.5)	318(37.8)		
**Age(years)**				7.79	0.020
18-25	716(75.3)	71(65.1)	645(76.6)		
26-35	222(23.3)	37(33.9)	185(22.0)		
≥36	13(1.4)	1(0.9)	12(1.4)		
**Legal marital status**				0.95	0.329
Never married	885(93.1)	99(90.8)	786(93.4)		
Ever married /engaged /widowed/divorced	66(6.9)	10(9.2)	56(6.7)		
**Highest educational attainment**				0.15	0.927
High school or below	235(24.7)	28(25.7)	207(24.6)		
Some college	285(30.0)	31(28.4)	254(30.2)		
Bachelor's or higher	431(45.3)	50(45.9)	381(45.3)		
**Annual income (USD)**					
< $2000	456(48.0)	50(45.9)	381(45.3)	8.67	0.034
$2000- $5000	253(26.6)	41(37.6)	212(25.2)		
$5000-$10000	87(9.1)	11(10.1)	76(9.0)		
> $10000	155(16.3)	16(14.7)	139(16.5)		
**Religion**				1.82	0.610
Christianity	812(85.4)	94(86.2)	718(85.3)		
Muslim	85(8.9)	8(7.3)	77(9.1)		
Other	8(0.8)	2(1.8)	6(0.7)		
None	46(4.8)	5(4.6)	41(4.9)		
**Reasons for migration**				4.83	0.185
Business	241(25.3)	31(28.4)	210(24.9)		
Study	629(66.1)	64(58.7)	565(67.1)		
Employment	58(6.1)	9(8.3)	49(5.8)		
Tourism/visiting relatives	23(2.4)	5(4.6)	18(2.1)		
**Cumulative stay in China**				2.75	0.253
1-6 months	25(2.6)	5(4.6)	20(2.4)		
7-12 months	33(3.5)	2(1.8)	31(3.7)		
One year and above	893(93.9)	102(93.6)	791(93.9)		
**Living arrangement in China**				16.75	0.005
Hotel	21(2.2)	6(5.5)	15(1.8)		

Guest apartment	32(3.4)	8(7.3)	24(2.9)		
Purchased apartment	10(1.0)	2(1.8)	8(1)		
Rental apartment	346(36.4)	44(40.4)	302(35.9)		
Staff/student dormitory	529(55.6)	47(43.1)	482(57.2)		
No regular residence	13(1.4)	2(1.8)	11(1.3)		
**Health insurance in China**				5.96	0.015
Yes	836(87.9)	88(80.7)	748(88.8)		
No	115(12.1)	21(19.3)	94(11.2)		
**Have you had sex during your stay in China**				5.23	0.022
Yes	367(38.6)	53(48.6)	314(37.3)		
No	584(61.4)	56(51.4)	528(62.7)		
**Regular partner***				1.69	0.194
Yes	341(92.9)	47(88.7)	294(93.6)		
No	26(7.1)	6(11.3)	20(6.4)		
**Casual partner***				1.23	0.289
Yes	40(10.9)	8(15.1)	32(10.2)		
No	327(89.1)	45(84.9)	282(89.8)		
**Number of sexual partners**					
	1.9±2.6	1.7±1.1	2.0±2.7	1.21	0.228
**Ever had commercial sexual activities**					
Yes	11(1.2)	3(2.8)	8(1)	2.74	0.098
No	940(98.8)	106(97.3)	834(99.1)		
**Consistently used condom in sexual activities**				0.05	0.831
Yes	185(50.4)	26(49.1)	159(50.6)		
No	182(49.6)	27(50.9)	155(49.4)		
**Injected drugs in the past year**				4.03	0.045
Yes	5(0.5)	2(1.8)	3(0.4)		
No	946(99.5)	107(98.2)	839(99.6)		
**Have you received health services in China in the past year†**				0.03	0.870
Yes	347(36.5)	39(35.8)	308(36.6)		
No	604(63.5)	70(64.2)	534(63.4)		
**Have you ever had any STD testing other than HIV testing ‡**				0.39	0.534
Yes	130(13.7)	17(15.6)	113(13.4)		
No	821(86.3)	92(84.4)	729(86.6)		
**Had HIV testing before coming to China**				1.69	0.194
Yes	898(94.4)	100(91.7)	798(94.8)		
No	53(5.6)	9(8.3)	44(5.2)		

HIV-human immunodeficiency virus, STD - sexually transmitted diseases.
* Participants had sex during the stay in China.
† Health services other than HIV testing.
‡ STD testing includes testing for syphilis, gonorrhea, chlamydia, human papilloma virus, and herpes simplex virus.

Table S8. Factors Correlated with mandatory HIV testing compared with voluntary testing among African migrants who ever had HIV testing in China, 2021 (*N*=951)

Variables	c*OR* (95%CI)	*P*	a*OR* (95%CI) §	*P*
**Gender**				
Male	*ref*		-	-
Female	0.93(0.62-1.40)	0.734	-	-
**Age(years)**				
16-25	*ref*		-	-
26-35	0.55(0.36-0.85)	0.007	-	-
≥36	1.32(0.17-10.31)	0.791	-	-
**Legal marital status**				
Never married	*ref*		-	-
Ever married/engaged	0.71(0.35-1.43)	0.331	-	-
**Highest educational attainment**				
High school or below	*ref*		-	-
Some college	1.11(0.64-1.91)	0.711	-	-
Bachelor’s or higher	1.03(0.63-1.69)	0.904	-	-
**Annual income (USD)**				
< $2000	*ref*		-	-
$2000- $5000	0.51(0.32-0.81)	0.005	-	-
$5000-$10000	0.68(0.34-1.39)	0.291	-	-
> $10000	0.86(0.47-1.58)	0.623	-	-
**Religion**				
Christianity	0.79(0.37-1.7)	0.551	-	-
Islam	0.31(0.05-1.81)	0.194	-	-
Other	0.85(0.26-2.77)	0.79	-	-
None	*ref*		-	-
**Reasons for migration**				
Business	*ref*		*ref*	
Study	1.30(0.83-2.06)	0.256	1.42(0.83-2.44)	0.202
Employment	0.80(0.36-1.80)	0.595	0.72(0.32-1.65)	0.44
Tourism/Visiting relatives	0.53(0.18-1.53)	0.242	0.52(0.17-1.56)	0.241
**Cumulative stay in China**				
1-6 months	*ref*		*ref*	
7-12 months	3.88(0.69-21.93)	0.126	5.10(0.87-30.05)	0.072
One year and above	1.94(0.71-5.28)	0.195	2.27(0.81-6.35)	0.12
**Living arrangement in China**				
Purchased apartment	*ref*		*ref*	
Hotel	1.20(0.35-4.15)	0.773	1.30(0.36-4.75)	0.692
Guest apartment	1.60(0.26-9.84)	0.612	1.04(0.16-6.84)	0.971
Rental apartment	2.75(1.01-7.45)	0.047	2.57(0.90-7.35)	0.079
Staff/student dormitory	4.1(1.52-11.08)	0.005	4.23(1.46-12.27)	0.008

No fixed residence	2.2(0.37-13.04)	0.385	2.32(0.37-14.52)	0.370
**Health insurance in China**				
Yes	0.53(0.31-0.89)	0.016	0.91(0.59-1.39)	0.657
No	*ref*		*ref*	
**Have you had sex during your stay in China**				
Yes	*ref*		*ref*	
No	1.59(1.07-2.38)	0.023	1.55(1.03-2.34)	0.036
**Regular partner***				
Yes	1.88(0.72-4.92)	0.2	1.98(0.69-5.69)	0.204
No	*ref*		*ref*	
**Casual partner***				
Yes	0.64(0.28-1.47)	0.293	0.52(0.21-1.28)	0.156
No	*ref*		*ref*	
**Number of sexual partners**				
	1.06(0.89-1.27)	0.489	1.04(0.87-1.25)	0.649
**Consistently used condom in sexual activities**				
Yes	*ref*		*ref*	
No	0.94(0.52-1.68)	0.832	0.95(0.52-1.75)	0.865
**Ever had commercial sexual activities**				
Yes	0.34(0.09-1.30)	0.114	0.39(0.09-1.58)	0.187
No	*ref*		*ref*	
**Injected drugs in the past year**				
Yes	5.23(0.86-31.64)	0.072	6.20(0.88-43.86)	0.068
No	*ref*		*ref*	
**Have you received health services in China in the past year†**				
Yes	0.97(0.64-1.46)	0.871	0.97(0.59-1.39)	0.657
No	*ref*		*ref*	
**Have you ever had any STD testing other than HIV testing‡**				
Yes	0.84(0.48-1.46)	0.534	0.86(0.49-1.52)	0.603
No	*ref*		*ref*	
**Had HIV testing before coming to China**				
Yes	1.63(0.77-3.44	0.198	1.68(0.78-3.64)	0.188
No	*ref*		*ref*	
**Acculturative stress**				
High	0.71(0.47-1.08)	0.112	0.70(0.45-1.06)	0.094
Moderate	0.37(0.17-0.81)	0.013	0.37(0.16-0.84)	0.018
Low	*ref*		*ref*	
**Feel Discriminated as a foreigner in China**				
High	0.68(0.45-1.04)	0.078	0.69(0.45-1.06)	0.089
Moderate	0.45(0.19-1.08)	0.074	0.48(0.20-1.18)	0.112
Low	*ref*		*ref*	
**Anticipated HIV stigma**				
High	1.35(0.88-2.08)	0.173	1.38(0.89-2.14)	0.156

Moderate	1.139(0.72-0.33)	0.331	1.50(0.76-2.94)	0.24
Low	*ref*		*ref*	
**Community engagement**				
High	0.64(0.38-1.08)	0.094	0.59(0.35-1.00)	0.052
Moderate	0.43(0.21-0.87)	0.019	0.40(0.19-0.85)	0.017

HIV-human immunodeficiency virus, aOR-adjusted OR, cOR-crude OR, CI-confidence interval.
* Participants had sex during the stay in China.
† Health services other than HIV testing.
‡ STD testing includes testing for syphilis, gonorrhea, chlamydia, human papilloma virus, and herpes simplex virus.
§Adjusted for gender, age, legal marital status, highest educational attainment, annual income, and religion.

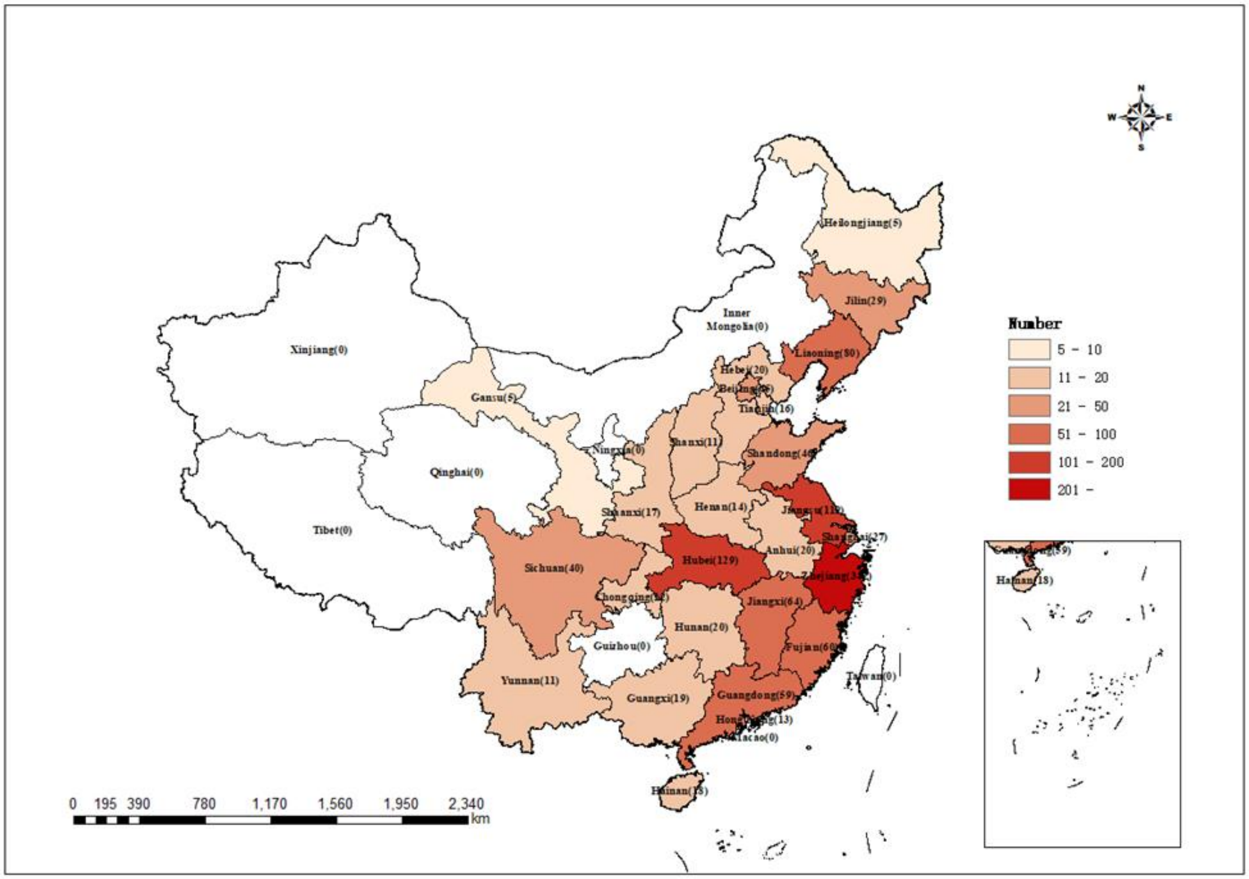

Figure S1 Geographic distribution of study participants in China (*N*=1305)
